# Low-Density Granulocytes Link to Disease Activity, Organ Involvement, and Cytokine Production in Sjögren’s Disease

**DOI:** 10.3390/ijms27156722

**Published:** 2026-07-28

**Authors:** Jing Ning, Yuebo Jin, Shiyu He, Bo Huang, Linger Guan, Jing He

**Affiliations:** Department of Rheumatology and Immunology, Beijing Key Laboratory of Non-Invasive Diagnosis and Immunotherapy for Rheumatic and Immune Diseases (BZ-2025-127), Peking University People’s Hospital, 11 Xizhimen South St, Beijing 100044, China

**Keywords:** low-density granulocytes, Sjögren’s disease, neutrophil

## Abstract

Low-density granulocytes (LDGs) have been implicated in the pathogenesis of several autoimmune diseases, yet their role in Sjögren’s disease (SjD) remains poorly understood. We enrolled 90 SjD patients and 30 healthy controls (HCs) and identified LDGs as CD14^−/low^CD15^+^ cells by flow cytometry, with intracellular interleukin-6 (IL-6) and tumor necrosis factor-alpha (TNF-α) staining, and further analyzed CD16 as a maturation marker in LDGs and T helper 17 (Th17) cell frequency. LDG percentages were significantly elevated in active SjD compared with inactive patients (*p* < 0.001) and HCs (*p* < 0.0001), and correlated positively with EULAR Sjögren’s Syndrome Disease Activity Index (ESSDAI) (r = 0.355, *p* = 0.0006), erythrocyte sedimentation rate (ESR) (r = 0.325, *p* = 0.0061), γ-globulin (r = 0.334, *p* = 0.0177), and Th17 frequency (r = 0.537, *p* = 0.0068). LDG expansion was accompanied by enrichment of immature CD16^−/low^ cells (r = −0.798, *p* = 0.0100). Patients with renal or pulmonary involvement showed higher LDG levels (*p* = 0.0004), and in SjD -associated interstitial lung disease (SjD-ILD) patients, LDG percentage showed a positive but non-significant trend with serum Krebs von den Lungen-6 (KL-6) levels (r = 0.497, *p* = 0.102). LDG levels decreased following treatment in longitudinally followed patients, and LDGs from active patients exhibited higher IL-6 and TNF-α production ratios relative to monocytes than those from inactive patients. Stratification by LDG levels revealed significant associations with disease activity, laboratory parameters, and organ involvement. These findings suggest that LDGs are associated with disease activity and organ involvement, may serve as potential biomarkers, and may contribute to the pathogenesis of SjD.

## 1. Introduction

Sjögren’s disease (SjD) is a systemic autoimmune disease characterized by lymphocytic infiltration of the exocrine glands, leading to xerostomia and xerophthalmia. Systemic manifestations occur in 30–40% of patients. Lymphocytic infiltration and immune complex deposition are the underlying mechanisms for organ damage beyond the exocrine glands. Organs commonly implicated are the pulmonary (11%), cutaneous (10%), renal (5%) and peripheral nervous system (6%). Such extraglandular manifestations contribute significantly to disease burden, morbidity, and mortality [1,2,3]. Despite advances in understanding the adaptive immune components of SjD, the precise mechanisms underlying systemic inflammation and organ damage remain incompletely defined, underscoring the need to explore additional immunopathogenic pathways.

Traditionally, SjD pathogenesis has been attributed to aberrant activation of B cells and T cells, resulting in autoantibody production, ectopic germinal center formation, and persistent inflammatory responses [4,5]. However, increasing evidence highlights the critical contribution of innate immunity to disease development and progression. Monocytes, macrophages, and plasmacytoid dendritic cells have all been implicated in amplifying inflammatory cascades and interacting with adaptive immune populations to perpetuate chronic inflammation [6,7,8]. In this context, neutrophils—long regarded primarily as terminal effector cells of innate immunity—are emerging as important regulators of autoimmunity [9].

Neutrophils contribute to the pathogenesis of several autoimmune diseases—such as systemic lupus erythematosus (SLE), Behçet’s disease, and adult-onset Still’s disease—by promoting Neutrophil extracellular trap formation (NETosis), degranulation, and producing cytokine, which exacerbate inflammation and tissue damage, thereby driving disease progression [10,11,12,13].

Among neutrophil subsets, low-density granulocytes (LDGs) have drawn increasing attention. LDGs are defined as neutrophils that aberrantly segregate within the peripheral blood mononuclear cell (PBMC) fraction after density gradient centrifugation [14]. Compared with normal-density neutrophils (NDNs), LDGs display a prominent type I interferon (IFN I) signature and are strongly associated with the activation of IFN-related pathways, along with enhanced pro-inflammatory cytokine production, and spontaneous NETosis [15,16]. Their pathogenic potential has been most extensively characterized in SLE, where elevated LDG levels correlate with disease activity, vascular injury, coronary artery disease, and lupus nephritis (LN) [16,17,18,19]. LDGs in SLE not only drive inflammation but also exhibit unique biophysical properties that may promote retention in microvasculature, contributing to tissue injury in highly vascularized organs [20].

In SjD, the role of neutrophils and LDGs remains underexplored. Early studies have suggested enhanced neutrophil activation and NETosis in both plasma and glandular tissues of SjD patients, with LDG frequencies reported to be elevated compared with healthy controls (HCs) [21]. However, the clinical relevance of LDG expansion in SjD and its potential mechanistic contribution to systemic disease manifestations remain largely unknown. Specifically, it is unclear whether LDG levels are associated with disease activity indices, systemic organ involvement such as renal or pulmonary complications, or inflammatory cytokine production. Moreover, the interplay between LDGs and adaptive immune subsets, such as T helper 17 (Th17) cells which are known to play critical pathogenic roles in SjD [5], has not been elucidated.

To address these gaps, we systematically investigated the prevalence and functional characteristics of LDGs in a well-defined cohort of SjD patients. We aimed to determine the associations of LDG frequencies with disease activity, systemic manifestations, and laboratory parameters, as well as to explore their contribution to cytokine production. Furthermore, we evaluated longitudinal changes in LDG levels following treatment to assess their potential as dynamic biomarkers. By delineating the clinical and immunological significance of LDGs in SjD, this study provides new insights into innate immune dysregulation and highlights LDGs as candidate biomarkers that may contribute to disease pathogenesis.

## 2. Results

### 2.1. Characteristics of Patients with SjD

A total of 90 patients with SjD were enrolled in our study. Exactly 86 (95.6%) patients were female, with a median age of 53 (38 to 61) years. The median disease duration was 5 (2 to 10) years, and the median EULAR Sjögren’s Syndrome Disease Activity Index (ESSDAI) score was 2.5 (1 to 6) points. Based on the ESSDAI, 35 (38.9%) patients had high disease activity and 55 (61.1%) had low disease activity. Regarding systemic manifestations, 11 (12.2%) patients had renal involvement and 13 (14.4%) had interstitial lung disease (ILD). Regarding treatment, many patients received combination therapy; thus, the following categories are not mutually exclusive. Overall, 32 patients (35.6%) received glucocorticoids (GC), 43 (47.8%) received hydroxychloroquine (HCQ), 49 (54.4%) received mycophenolate mofetil (MMF), eight (8.9%) received cyclosporin A (CsA), five (5.6%) received iguratimod, seven (7.8%) received Janus kinase (JAK) inhibitors, and five (5.6%) received biologic agents. The most frequent co-medication pairs were HCQ plus MMF and glucocorticoids plus MMF (*n* = 22, 24.4% each), followed by glucocorticoids plus HCQ (*n* = 17, 18.9%).

### 2.2. Elevated LDG Levels in SjD and Correlation with Disease Activity

**Figure 1:**
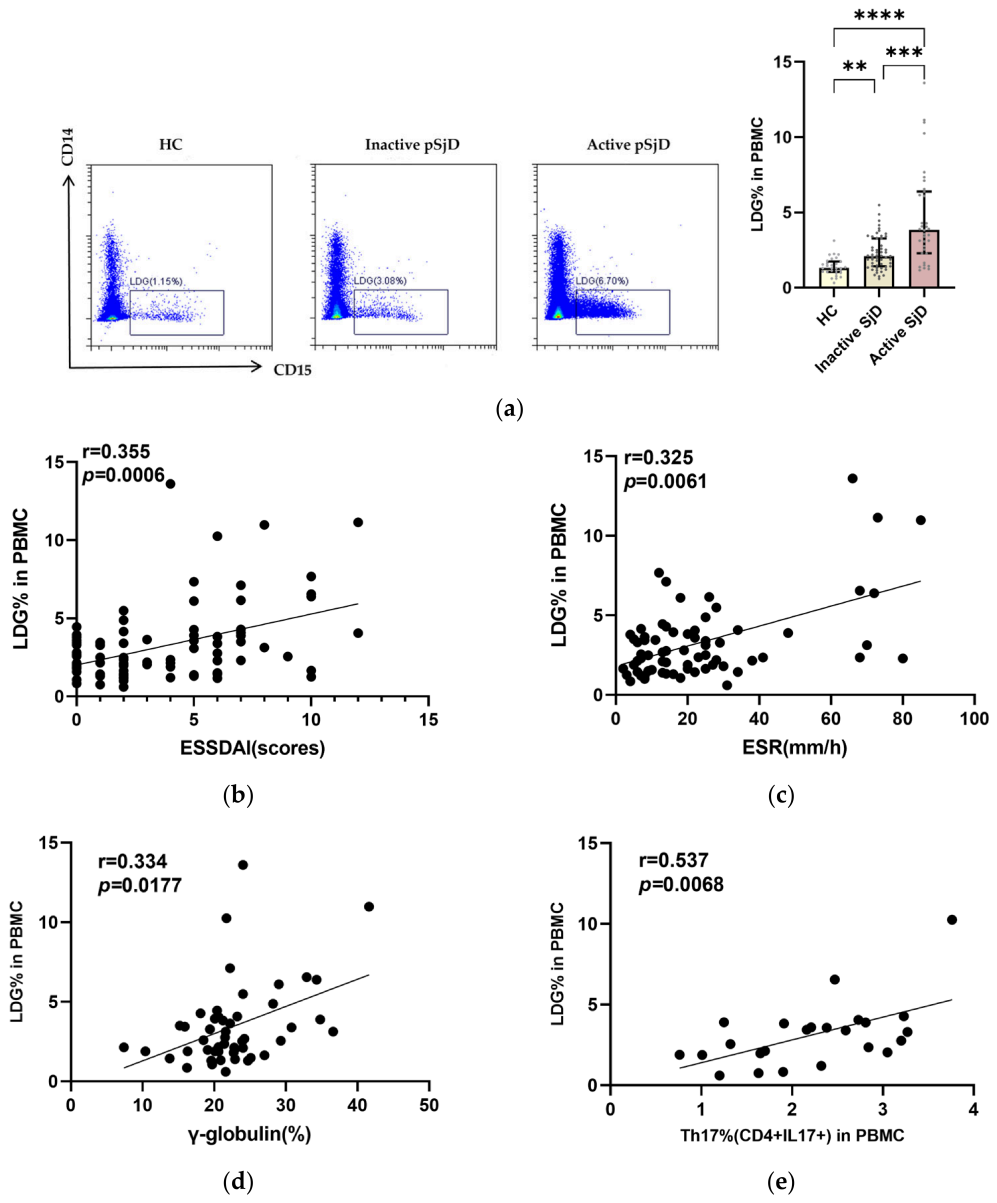
Association of LDG proportion in PBMCs with disease activity, laboratory parameters, and Th17 cell levels in SjD patients. (**a**) Representative flowcytometry results and comparison of LDG% in PBMC in patients with active SjD (*n* = 35), patients with inactive SjD (*n* = 55) and HCs (n = 30); LDG% in PBMC were positively correlated with ESSDAI scores (n = 90) (**b**), ESR (mm/h) (n = 71) (**c**), γ-globulin (%) (*n* = 50) (**d**) and Th17% in PBMC (*n* = 24) (**e**). Data are presented as median with interquartile range. Kruskal–Wallis test followed by Dunn’s multiple comparisons test, Spearman Correlation. ** *p*-value < 0.01, *** *p*-value < 0.001, **** *p*-value < 0.0001. *p*-values are nominal and have not been adjusted for study-wide multiple comparisons; therefore, secondary and subgroup findings should be interpreted as exploratory. Abbreviations: LDG, low-density granulocyte; PBMC, peripheral blood mononuclear cell; Th17, T helper 17; SjD, Sjögren’s disease; HC, healthy control; ESSDAI, EULAR Sjögren’s Syndrome Disease Activity Index; ESR, erythrocyte sedimentation rate.

We analyze the association of LDG levels with disease activity indices in SjD. We found that the percentage of LDGs among PBMCs were markedly elevated in patients with active SjD compared to those with inactive disease (*p* < 0.001) and HCs (*p* < 0.0001, Figure 1a). The median LDG percentage was 3.83% [2.31–6.39] in active SjD patients, significantly higher than the 2.11% [1.43–3.28] observed in inactive patients and 1.31% [1.05–1.75] in HCs. Consistent with this finding, LDG% in PBMC was significantly correlated with the ESSDAI score in patients with SjD (*n* = 90, r = 0.355, *p* = 0.0006, Figure 1b). For laboratory parameters, the LDG percentage showed significant positive correlations with the erythrocyte sedimentation rate (ESR) (*n* = 71, r = 0.325, *p* = 0.0061, Figure 1c) and γ-globulin levels (*n* = 50, r = 0.334, *p* = 0.0177, Figure 1d). In the subset of 24 patients evaluated for Th17 cells, LDG percentage also correlated positively with the proportion of Th17 cells in PBMCs (*n* = 24, r = 0.537, *p* = 0.0068; Figure 1e), suggesting a potential link between LDG expansion and Th17-mediated adaptive immunity in SjD.

**Figure 2:**
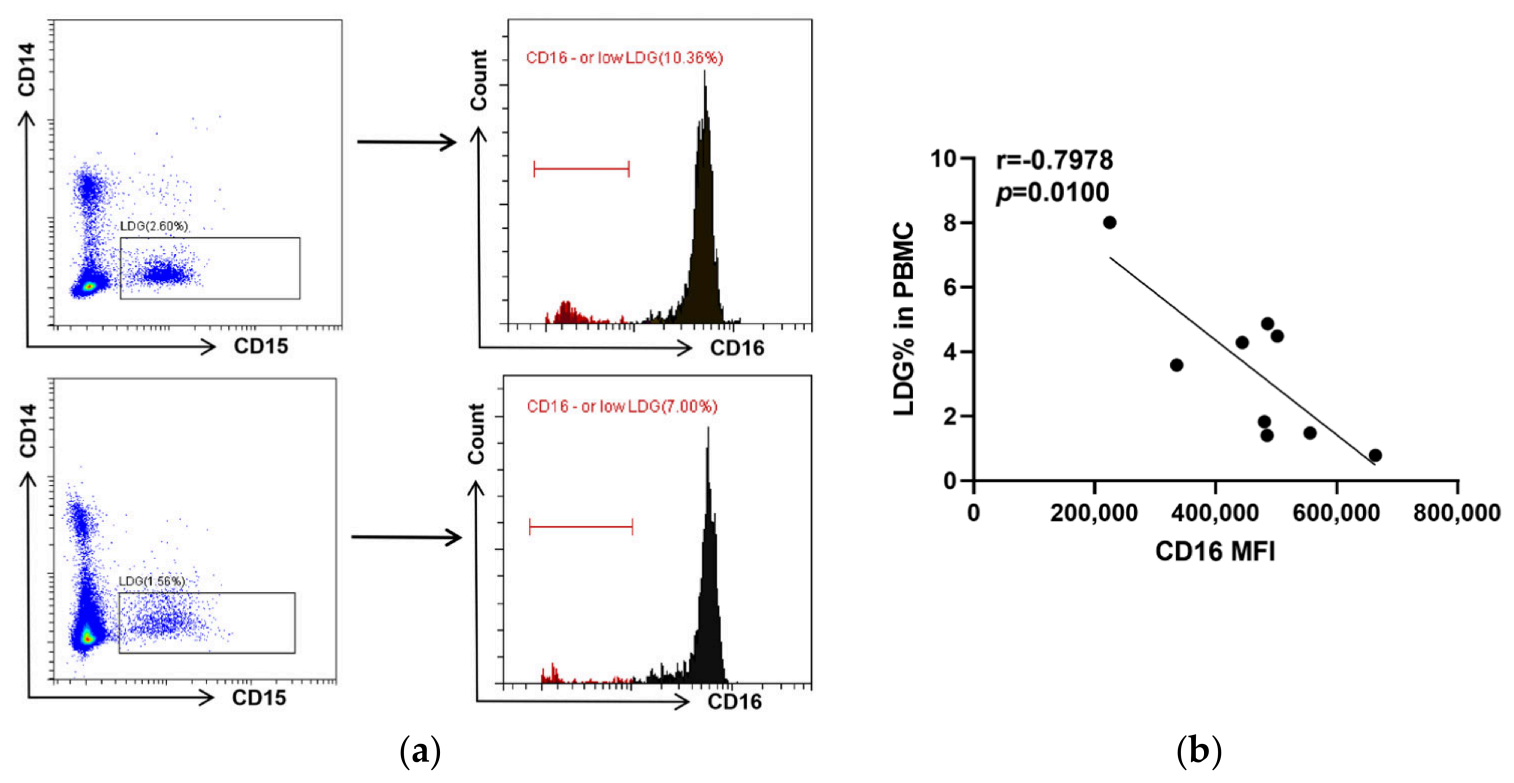
The proportion of immature LDGs (CD16^−/low^ LDG) increases with LDG%. (**a**) Representative flow cytometry results of CD16 expression in patients with different LDG%. (**b**) The CD16 MFI is negatively correlated with the percentage of LDGs within PBMC (*n* = 9, independent validation cohort). Pearson Correlation. Abbreviations: LDG, low-density granulocyte; MFI, mean fluorescence intensity; PBMC, peripheral blood mononuclear cell.

To characterize the maturation status of LDGs, we assessed CD16 expression in an independent cohort of nine patients with SjD. CD16 mean fluorescence intensity (MFI) on LDGs was negatively correlated with LDG% in PBMCs (r = −0.798, *p* = 0.0100, Figure 2), indicating that LDG expansion was accompanied by a relative enrichment of immature CD16^−/low^ cells.

### 2.3. Increased LDGs Were Correlated with Renal and Pulmonary Involvements in Patients with SjD

**Figure 3:**
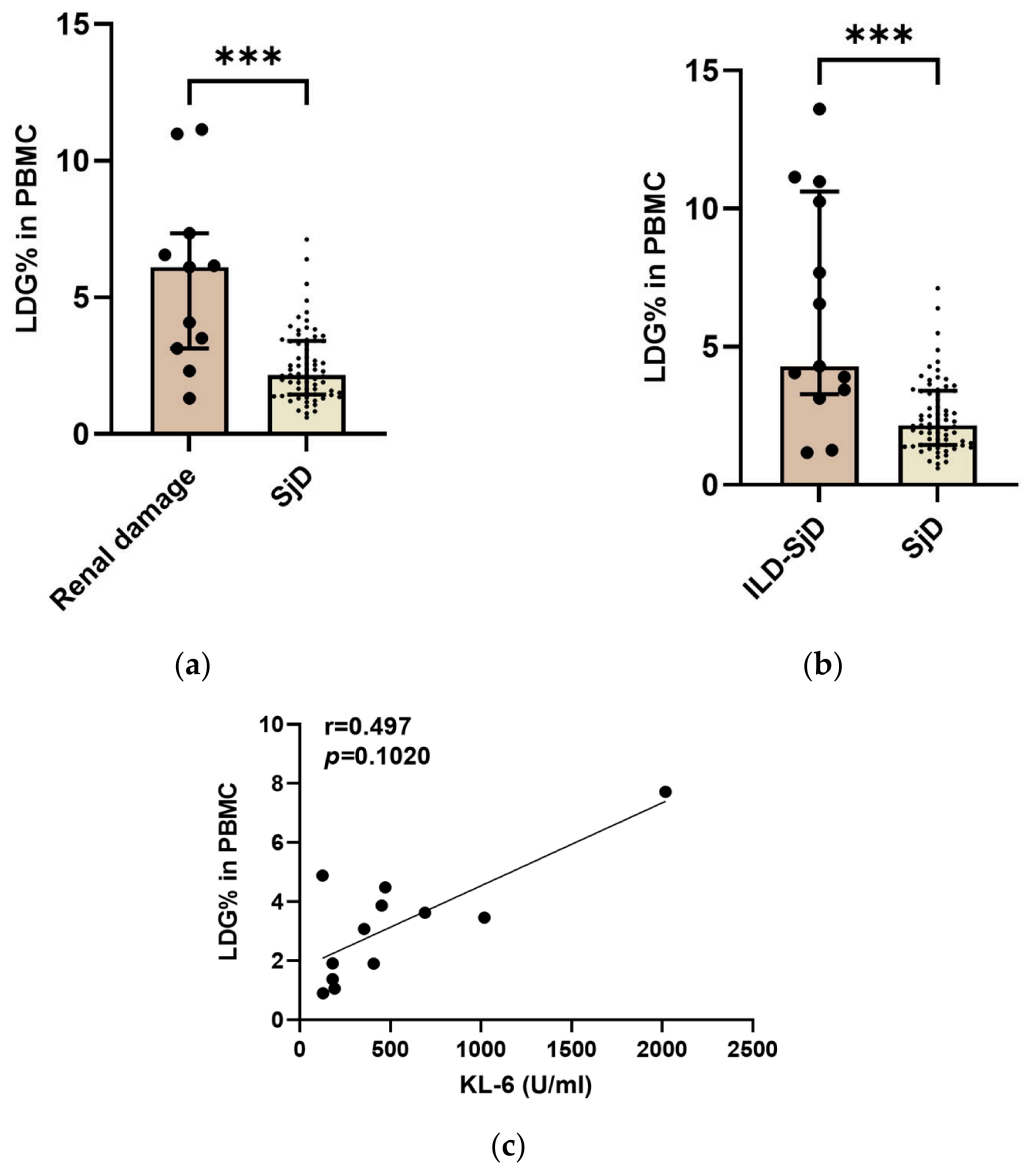
Association of elevated LDG levels with renal and pulmonary involvement in patients with SjD. Patients with SjD who had renal damage (n = 11) (**a**) or ILD (n = 13) (**b**) had higher LDG percentages than the comparator group (n = 71), which comprised patients with no clinically evident pulmonary, renal, cutaneous, articular, or peripheral nervous system involvement. Mann–Whitney U test, *** *p*-value < 0.001. (**c**) Relationship between serum KL-6 levels and the percentage of LDGs in PBMCs from patients with SjD-ILD (n = 12; independent validation cohort). Spearman Correlation. Abbreviations: LDG, low-density granulocyte; SjD, Sjögren’s disease; ILD, interstitial lung disease; KL-6, Krebs von den Lungen-6; PBMC, peripheral blood mononuclear cell; SjD-ILD, Sjögren’s disease-associated interstitial lung disease.

Given that the kidney and lungs are frequently involved in SjD, we next investigated whether LDG levels are associated with these organ complications. Analysis revealed that patients with these complications exhibited significantly elevated LDG percentages compared to those without. Specifically, the mean LDG percentage was markedly higher in patients with renal involvement (*n* = 11, 6.11% [3.13–7.34], *p* = 0.0004; Figure 3a) and in those with ILD (*n* = 13, 4.29% [3.29–10.62], *p* = 0.0004; Figure 3b) than in patients without clinically evident extraglandular organ involvement (i.e., without pulmonary, renal, cutaneous, articular, or peripheral nervous system involvement, *n* = 71, 2.16% [1.45–3.40]). To further investigate the relationship between LDGs and pulmonary involvement in SjD, serum KL-6 (Krebs von den Lungen-6) levels were collected from 12 SjD-ILD patients (an independent validation set, not drawn from the main cohort of 90 patients). KL-6 is a sialylated glycoprotein antigen that reflects alveolar epithelial injury and serves as a widely used biomarker for assessing ILD activity [22]. LDG frequency showed a positive but non-significant trend with serum KL-6 levels (r = 0.497, *p* = 0.102; Figure 3c). Given the small sample size, this trend should be considered exploratory and warrants further investigation in a larger independent cohort.

### 2.4. Longitudinal Changes in LDG Levels After Treatment

**Figure 4:**
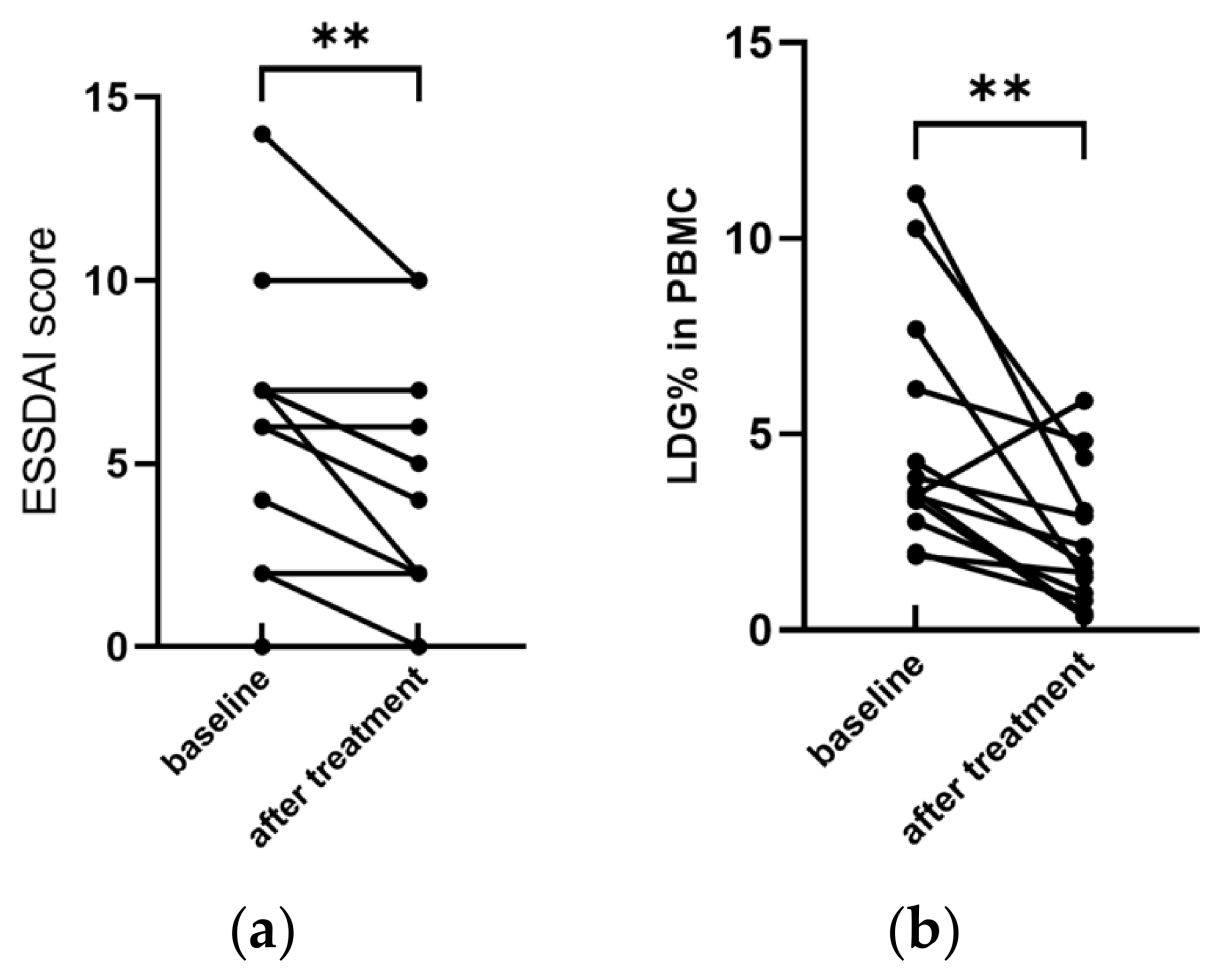
Reduction in LDGs in patients with SjD following treatment. Following 3–6 months of treatment, 13 SjD patients from the main cohort showed reductions in both ESSDAI scores (**a**) and LDG levels (**b**). Data are presented as median with interquartile range. Wilcoxon test, ** *p*-value < 0.01. Abbreviations: LDG, low-density granulocyte; SjD, Sjögren’s disease; ESSDAI, EULAR Sjögren’s Syndrome Disease Activity Index.

To assess the dynamics of LDG levels in response to therapy, we longitudinally followed 13 patients (nine with active disease and four with inactive disease) and measured their LDG percentages at baseline and after a 3–6-month treatment period. During follow-up, patients received individualized treatment according to disease activity and organ involvement. The medications included HCQ (*n* = 10), GC (*n* = 6), MMF (*n* = 6), CsA (*n* = 2), and biologic agents (rituximab [RTX] *n* = 2; belimumab [BEL], *n* = 1). Detailed treatment regimens are provided in Supplementary Table S1. After 3–6 months of treatment, ESSDAI scores significantly decreased (*p* < 0.01, Figure 4a), accompanied by a significant reduction in LDG percentages compared with baseline (*p* < 0.01, Figure 4b). These findings further support an association between LDG levels and disease activity in SjD.

### 2.5. Elevated Cytokine Production by LDGs in Patients with Active SjD

**Figure 5:**
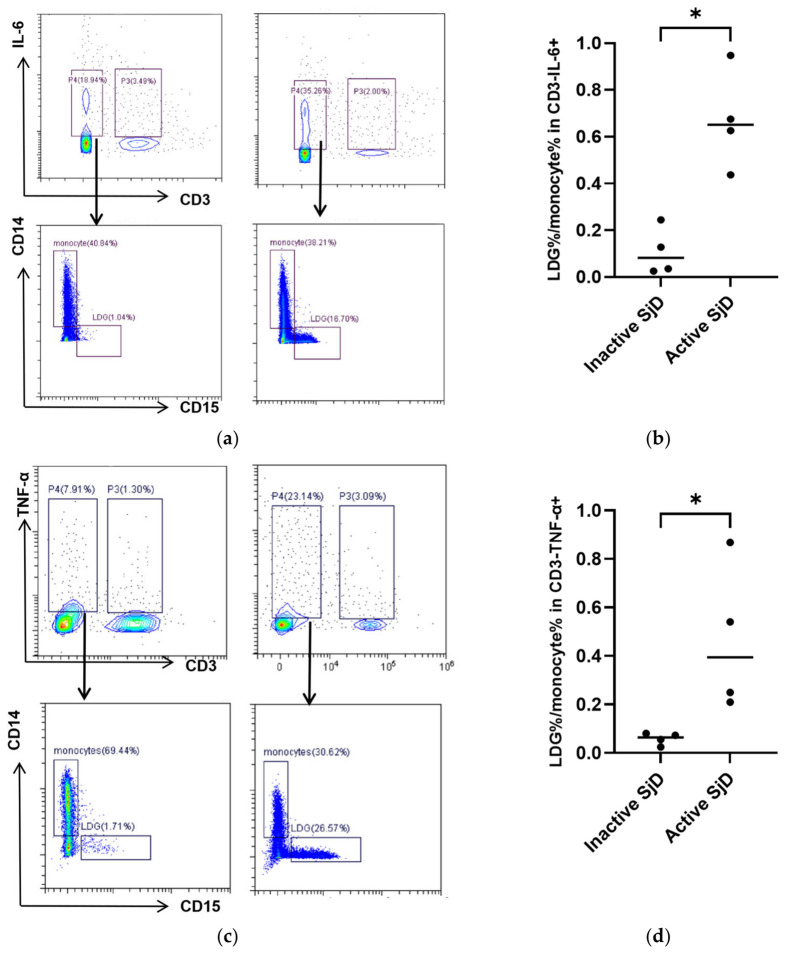
Elevated cytokine production by LDGs in patients with active SjD. Intracellular IL-6 expression in LDGs and CD14+ monocytes from SjD patients stratified by disease activity. (**a**) Representative flow results for IL-6-producing LDGs. (**b**) Comparison of LDG-to-CD14^+^ monocyte ratio of CD3^−^IL-6-producing cells in active SjD (*n* = 4) and inactive SjD (*n* = 4). (**c**) Representative flow results for TNF-α–producing LDGs and representative flow plots for CD3^−^TNF-α-producing LDGs (**d**) Comparison of LDG-to-CD14^+^ monocyte ratio of CD3^−^TNF-α-producing cells in active SjD (*n* = 4) and inactive SjD (*n* = 4). Data are presented as median with range. Mann–Whitney U test; * *p* < 0.05. Abbreviations: LDG, low-density granulocyte; SjD, Sjögren’s disease; IL-6, interleukin-6; TNF-α, tumor necrosis factor-alpha.

To assess the cytokine-producing profile of LDGs in SjD, intracellular tumor necrosis factor-alpha (TNF-α) and interleukin-6 (IL-6) production was analyzed in LDGs and monocytes from four active and four inactive patients with SjD. We performed intracellular staining of lipopolysaccharide (LPS) stimulated PBMCs and compared the proportions of IL-6- and TNF-α-producing LDGs and monocytes among patients with different disease activity levels. In patients with inactive SjD, CD14^+^ monocytes represented the dominant source of IL-6 among CD3^−^IL-6^+^ PBMCs (range, 31.45–56.37%), while LDGs contributed only a minor fraction (0.81–13.26%). In active SjD, monocytes remained the predominant IL-6-producing population (14.46–44.83%); yet, LDG contribution rose markedly to 9.06–27.66%, reflecting a relative shift in cellular sources with increasing disease activity. A parallel pattern was observed for TNF-α: monocytes accounted for 27.60–69.44% in inactive versus 30.62–57.51% in active patients, while LDG-derived TNF-α increased substantially from 0.84 to 3.18% to 8.91–28.33%. To further evaluate the relative contribution of LDGs to cytokine production, we calculated the ratios of cytokine-producing LDGs to CD14^+^ monocytes, as described in earlier studies [23]. The LDG/CD14^+^ monocyte ratio was significantly elevated in active SjD group for both IL-6 (0.44–0.95 vs. 0.03–0.24; *p* < 0.05, Figure 5a,b) and TNF-α (0.21–0.87 vs. 0.03–0.08; *p* < 0.05, Figure 5c,d) compared to the inactive SjD group.

### 2.6. Clinical Stratification by LDG Levels

To further evaluate the clinical relevance of LDGs in SjD, 90 patients were categorized into low-LDG (*n* = 39) and high-LDG (*n* = 51) groups using a cut-off of 2.21% (90th percentile of LDG levels in the HC group). Disease activity, laboratory parameters, and systemic manifestations were compared between the two groups. Significant differences were observed in ESSDAI scores, ESR, γ-globulin levels, as well as in renal and pulmonary involvement. In contrast, no statistically significant between-group differences were observed in the use of GC, HCQ, MMF, CsA, iguratimod, JAK inhibitors, or biologic agents at the time of blood sampling (Table 1).

## 3. Discussion

LDGs are recognized for their pro-inflammatory roles in multiple autoimmune diseases; however, their function and clinical relevance in SjD remain incompletely characterized. In the present study, we demonstrate for the first time that LDG levels correlate significantly with disease activity and organ-specific complications in SjD, and that LDGs from patients with active disease exhibit increased pro-inflammatory cytokine production. Furthermore, a positive correlation was observed between LDG levels and the frequency of Th17 cells, suggesting an association between LDGs and adaptive immunity in SjD that needs further investigation.

Prior studies have established that elevated LDG levels correlate with disease activity across several autoimmune conditions [19,23,24], and our findings extend this association to SjD. Mechanistically, prior studies have demonstrated that LDGs possess enhanced NETosis capability compared with normal-density neutrophils [15]. In light of this, elevated LDGs may represent an important cellular source contributing to the increased NETosis markers previously reported by Yu Peng et al. to correlate with disease activity in SjD [21], further linking LDG biology to established pathogenic pathways in this disease.

Accumulating studies found that LDGs are implicated in systemic involvement in SLE [17,18]; however, whether LDG levels correlate with organ-specific complications in SjD has remained unclear. In the present study, we observed that SjD patients with either pulmonary or renal involvement exhibited significantly elevated LDG levels compared to those without organ complications. With respect to pulmonary involvement, LDG frequency showed a moderate positive trend with serum KL-6 levels, a known marker of type II pneumocyte damage and regeneration [22]; however, this association did not reach statistical significance (r = 0.497, *p* = 0.102). Therefore, the relationship between LDGs and the severity of pulmonary involvement remains uncertain. Nevertheless, previous experimental evidence provides a biological rationale for further investigation, as neutrophil elastase and NETs have been shown to activate transforming growth factor-beta (TGF-β) and promote fibrogenesis in experimental ILD models [25]. Moreover, in SjD-ILD, the neutrophil-to-lymphocyte ratio (NLR) has been reported to rise as early as three months before the onset of acute exacerbation, a significant decline in pulmonary function [26]. This finding further supports that neutrophils may be involved in the progression of SjD-ILD. As for other autoimmune disease, in dermatomyositis (DM), a study has shown that the LDG percentage was 2.7-fold higher in DM patients with ILD than those without, and positively correlated with lung disease activity scores [27]. Similarly, in rheumatoid arthritis, immunosuppressive phenotype as polymorphonuclear myeloid-derived suppressor cells (PMN-MDSCs) (HLA-DR^−^CD14^−^CD16^+^ CD15^+^CD33^mid^) in RA-ILD is significantly higher than in those without [28]. Collectively, these findings underscore the need to further elucidate the pathophysiological role of LDGs in connective tissue disease-associated ILD. Regarding renal involvement, Tay et al. [17] reported that the neutrophil-to-platelet ratio (NPR), correlates with LDN levels and is associated with LN activity, leading the authors to propose that LDNs may play a critical role in the development of nephritis. Underpinning the association between LDGs and both pulmonary and renal complications is likely their distinctive biophysical properties: LDGs isolated from SLE patients display a rougher cell surface compared with NDNs, facilitating their preferential retention within the microvasculature and potentially promoting endothelial injury and tissue damage in these highly vascularized organs [20].

The positive correlation between LDG frequency and Th17 cell abundance in PBMCs from SjD patients represents a notable finding that warrants further mechanistic investigation. In SjD, Th17 cells play a critical pathogenic role by promoting tissue injury, driving autoantibody production, secreting pro-inflammatory cytokines, and forming ectopic germinal centers [5]. Several studies indicate that LDGs can influence T cell differentiation and function. For instance, LDGs from patients with SLE significantly induce Th1-type pro-inflammatory cytokines [19] and myeloid-derived suppressor cells (MDSCs) from patients with SLE promote Th17 cell differentiation through an arginase-1-dependent mechanism [29]. Our study revealed a positive correlation between the LDG proportion and the frequency of Th17 (CD4^+^IL-17A^+^) cells in the PBMCs of patients with SjD, suggesting a potential interaction between these cell populations. Distinguishing between these possibilities will require co-culture experiments and longitudinal studies that can establish the temporal sequence of LDG and Th17 changes relative to disease flares.

Elevated levels of IL-6 and TNF-α have been documented in both the saliva and serum of SjD patients [30,31,32]. Monocytes are considered a key cellular source of these cytokines [33], and notably, IL-6 derived from monocytes contributes to the pathogenesis of SjD by promoting B cell hyperactivation and autoantibody production [34]. In our study, we revealed a significant expansion in the proportion of IL-6- and TNF-α-producing LDGs relative to monocytes in patients with active SjD, even though monocytes were still the dominant source. These findings suggest that LDGs may represent an additional source of pro-inflammatory cytokines in SjD.

The functional identity of the LDG population requires careful consideration in the context of the broader low-density neutrophil (LDN) literature. LDNs represent a heterogeneous population whose phenotype is context-dependent: in cancer, pregnancy, and infection, LDNs predominantly adopt an immunosuppressive phenotype as PMN-MDSCs; whereas, in autoimmune diseases they are generally regarded as pro-inflammatory LDGs [9]. These two populations demonstrate substantial immunophenotypic overlap, with PMN-MDSCs defined as HLA-DR^−^CD11b^+^CD14^−^CD15^+^CD33^mid^ [35], and LDGs characterized as CD14^−/low^CD15^+^ [19], consequently, functional T cell suppression assays are required for their definitive distinction [9]. However, the nomenclature for LDNs in autoimmune diseases remains inconsistent, partly due to limited understanding of their functional roles, with some studies classifying them as PMN-MDSCs. In the present study, we did not perform T cell suppression assays, and therefore cannot formally exclude the presence of PMN-MDSCs within our LDG gate. Nevertheless, the pro-inflammatory functional profile observed in our cohort, including enhanced cytokine production and a positive correlation with disease activity indices and inflammatory markers, is more consistent with an LDG than a PMN-MDSC phenotype. The coexistence of pro-inflammatory and immunosuppressive functions within this population may be partly explained by phenotypic plasticity. Evidence from murine models of Sjögren’s syndrome (experimental Sjögren’s syndrome, ESS) suggests that MDSCs may undergo a functional transition during disease progression: early-stage MDSCs retain suppressive capacity, but with disease advance, this function is lost alongside declining arginase-1 activity and glucocorticoid-induced tumor necrosis factor receptor-related ligand (GITRL) expression increases. Notably, in vitro stimulation experiments demonstrated that GITRL ligand-activated MDSCs enhanced Th1/Th17 immune responses, suggesting a potential shift toward a pro-inflammatory phenotype [36] though whether this transition occurs in vivo and in human SjD remains to be confirmed. Defective aryl hydrocarbon receptor (AhR) signaling has additionally been implicated in the impaired immunosuppressive function of PMN-MDSCs in ESS [37]. Whether LDGs in SjD undergo an analogous functional transition across disease stages remains an open question that longitudinal studies with paired functional assessments could address.

This study has several limitations. First, the sample sizes for the CD16 maturation, cytokine production, KL-6, and subgroup analyses (e.g., renal and ILD involvement) were relatively small; therefore, these findings should be considered preliminary and require validation in larger independent cohorts. Second, although a subset of patients underwent longitudinal follow-up, the study was predominantly cross-sectional, limiting causal inference regarding the relationship between LDG expansion and disease progression. Third, the longitudinal cohort was small and predominantly comprised patients with active disease at baseline (9 of 13 patients); therefore, regression to the mean may have contributed to the observed decline in LDG levels. The treatment regimens were also heterogeneous, precluding attribution of this decline to any specific treatment. These findings should therefore be interpreted as a longitudinal association rather than evidence of a treatment effect and require validation in larger prospective cohorts. Fourth, mechanistic experiments were limited to cytokine production, and further studies are needed to clarify the pathways through which LDGs may influence adaptive immunity, tissue infiltration, and organ injury. Fifth, because LDGs are heterogeneous, we did not further characterize distinct LDG subsets using additional markers of neutrophil maturation and activation. Sixth, no formal adjustment for multiple testing was prespecified because the analyses were exploratory. Accordingly, the reported *p* values are nominal, and the number of comparisons may have increased the risk of type I error. Secondary, subgroup, and borderline findings should therefore be regarded as hypothesis-generating. In particular, the KL-6 result represents a non-significant exploratory trend rather than evidence of an association and requires confirmation in a larger independent cohort. Finally, the lack of adjustment for potential treatment-related confounders may have resulted in residual confounding, while the single-center design may limit the generalizability of our findings.

## 4. Materials and Methods

### 4.1. Patient Enrolment

A total of 119 patients diagnosed with SjD according to the 2002 American–European Consensus Group (AECG) classification criteria [38] were recruited from the Department of Rheumatology and Immunology at Peking University People’s Hospital between October 2024 and July 2025. Although the 2002 AECG criteria were used for initial enrollment, all patients also fulfilled the 2016 ACR/EULAR classification criteria upon retrospective re-evaluation. Of these, 90 patients constituted the main cohort for primary analyses, together with 30 age- and sex-matched HCs. In addition, three independent validation subsets were enrolled for specific analyses: an additional 9 SjD patients for assessment of LDG maturation status, and an additional 12 SjD patients with interstitial lung disease (ILD) for measurement of serum KL-6 levels, and 8 patients with SjD for intracellular IL-6 and TNF-α production analyses, comprising 4 patients with active SjD and 4 with inactive SjD. There was no participant overlap between the main cohort and these three independent cohorts or among the three independent cohorts.

For the main cohort of 90 patients, following laboratory testing and physician-conducted clinical evaluation, demographic parameters and disease-relevant data were respectively collected. For variables with incomplete data collection, analyses were conducted among participants with available data, and the analytic sample size is reported for each analysis. Disease activity was assessed by the Sjögren’s Syndrome Disease Activity Index (ESSDAI), with a score of ≥5 defining active disease.

Renal involvement in SjD was defined as previously described [39], which included any of the following: a compatible renal biopsy, significant urinalysis abnormalities (leukocyturia, haematuria, or casts), persistent proteinuria (>500 mg/24 h), renal tubular acidosis (with or without hypokalaemia), or recurrent nephrolithiasis with acidosis. Pulmonary involvement is defined by the manifestation of interstitial lung disease on high-resolution computed tomography (CT) or abnormal pulmonary function tests, defined as a diffusing capacity for carbon monoxide (DLCO) of less than 70% or a forced vital capacity (FVC) of less than 80%. Moreover, thirteen patients (9 active, 4 inactive) from the main cohort underwent a follow-up assessment after 3–6 months of treatment. They were not added again when calculating the total number of patients. The study was approved by the Ethics Committee of Peking University People’s Hospital (protocol code 2023PHB093-001), and all participants provided written consent to the study.

### 4.2. Identification of LDGs in PBMC Fractions

Peripheral blood collected in ethylenediaminetetraacetic acid (EDTA) tubes was diluted 1:1 with phosphate-buffered saline (PBS). The diluted blood underwent density gradient centrifugation, 1800 rpm, 20 min over Lymphocyte Separation Medium (TBDscience, Tianjin, China). PBMC layer at the interface was collected and washed with PBS. Low-density granulocytes (LDGs) within the PBMC fraction were quantified by a CytoFLEX flow cytometer (Beckman Coulter, Indianapolis, IN, USA). The gating strategy was as follows: cells were first selected based on forward scatter (FSC) and side scatter (SSC) to exclude debris, followed by singlet discrimination, and then gating on the CD14^−/low^CD15^+^ population using FITC-CD14 and PerCP-Cy5.5-CD15 antibodies (both from BioLegend, San Diego, CA, USA) (Figure S1). This definition of LDGs as CD14^−^/lowCD15^+^ cells within the PBMC fraction is consistent with the seminal work by Denny et al. [16] and has subsequently been widely adopted in studies of LDGs across autoimmune diseases. APC-CD16 (BioLegend, San Diego, CA, USA) was additionally used to assess LDG maturation status.

### 4.3. Intracellular Staining in Human PBMCs

For intracellular cytokine staining of LDGs, PBMCs were seeded at 1 × 10^6^ cells per well in 24-well plates and stimulated with LPS (1 μg/mL, Sigma-Aldrich, Burlington, MA, USA) for 4 h at 37 °C in 5% CO_2_. Monensin (BD GolgiStop™, BD Biosciences, Franklin Lakes, NJ, USA) was added at the onset of stimulation to block protein transport, as previously described [23]. Unstimulated cells cultured in medium alone served as negative controls. After stimulation, cells were harvested and surface-stained with PerCP-Cy5.5-CD15, FITC-CD14, and APC-Cy7-CD3 (all from BioLegend, San Diego, CA, USA) for 30 min on ice. Cells were then fixed and permeabilized using the Cytofix/Cytoperm™ Kit (BD Biosciences), followed by intracellular staining with PE-IL-6 and APC-TNF-α (both from BioLegend) for 30 min at 4 °C. Cells were washed twice with Perm/Wash buffer and resuspended in staining buffer for flow cytometric acquisition. LDG-derived IL-6 and TNF-α were analyzed within the CD3^−^CD14^−/low^CD15^+^ gate.

For Th17 cell detection, PBMCs were resuspended in complete RPMI-1640 medium at 1–2 × 10^6^ cells/mL and stimulated with Cell Activation Cocktail (with Brefeldin A) (500×, BioLegend, Cat. No. 423303) at 2 μL/mL for 5 h at 37 °C in 5% CO_2_. Unstimulated controls were processed in parallel. After stimulation, cells were centrifuged, washed with PBS, and surface-stained with APC-Cy7-CD3, FITC-CD4 (all from BioLegend) for 30 min at room temperature. Cells were then fixed and permeabilized with BD Cytofix/Cytoperm™ solution (250 μL) for 30 min at 4 °C, washed twice with Perm/Wash buffer, and intracellularly stained with BV421-IL-17A (BioLegend) for 30 min at 4 °C. After washing, cells were resuspended in staining buffer and acquired on a CytoFLEX flow cytometer. Th17 cells were defined as CD4^+^IL-17A^+^ within the lymphocyte population. Unstimulated cells cultured in medium alone served as negative controls for both assays.

### 4.4. Statistical Analysis

Continuous variables are presented as mean ± standard deviation (SD) or median with interquartile range (IQR) as indicated in the figure legends. For small sample comparisons (*n* ≤ 5 per group), data are presented as median (range) rather than interquartile range. Categorical variables are summarized as frequency (percentage). Group comparisons were performed using the Mann–Whitney U test; whereas, paired comparisons were analyzed using the Wilcoxon signed-rank test. Comparisons among three groups were performed using the Kruskal–Wallis test, followed by Dunn’s multiple comparisons test for pairwise comparisons when appropriate. Categorical variables were compared using the Chi-square (χ^2^) test or Fisher’s exact test when expected cell counts were <5. Correlations were assessed using Pearson’s correlation analysis for normally distributed variables and Spearman’s correlation analysis for non-normally distributed variables. All analyses were conducted using SPSS (version 27.0; IBM, Armonk, NY, USA) or GraphPad Prism (version 5.0; GraphPad Software, San Diego, CA, USA). A two-tailed *p*-value less than 0.05 was considered statistically significant.

## 5. Conclusions

This study provides the first report establishing a significant association between LDG levels and disease activity, systemic involvement, and pro-inflammatory cytokine production in SjD. These findings suggest that LDGs may serve as potential biomarkers for disease activity and systemic involvement, and may contribute to disease pathogenesis. Further mechanistic studies are warranted to clarify their role in the interplay between innate and adaptive immunity in SjD.

## Figures and Tables

**Table 1:** Clinical characteristics of SjD patients stratified by LDG levels.

Variables	Low LDGs (*n* = 39)	High LDGs (*n* = 51)	*p*-Value
Demographic characteristics			
Gender, female	37 (94.9)	49 (96.1)	1.000
Age, years	54 (39, 58)	51 (38, 62)	0.765
Disease duration, years	5 (2, 9)	5 (2, 10)	0.687
Systemic involvements			
Pulmonary	2 (5.1)	11 (21.6)	0.028 *
Renal	1 (2.6)	10 (19.6)	0.034 *
Hematological			
Lymphocytopenia	5 (12.8)	5 (9.8)	0.910
Thrombocytopenia	3 (7.7)	2 (3.9)	0.757
Anemia	0 (0)	5 (9.8)	0.122
Laboratory parameters			
γ-globulin, %	20.6 (17.7, 22.9)	22.20 (20.45, 29.23)	0.040 *
Immunoglobulin G, g/L	16.61 (13.77, 19.13)	16.77 (12.59, 21.04)	0.710
Erythrocyte sedimentation rate, mm/h	13.5 (7.52, 24.25)	22 (11.75, 35.75)	0.029 *
Anti-SSA positive	34 (87.2)	46 (90.2)	0.741
Anti-SSB positive	15 (38.5)	27 (52.9)	0.348
Disease activity index			
ESSDAI score	2 (1, 4)	5 (1, 7)	0.010 **
Treatments			
Glucocorticoids	11 (28.2)	21 (41.2)	0.203
Hydroxychloroquine	16 (41.0)	27 (52.9)	0.293
Mycophenolate mofetil	21 (53.8)	28 (54.9)	0.921
Cyclosporine A	5 (12.8)	3 (5.9)	0.286
Iguratimod	1 (2.6)	4 (7.8)	0.384
JAK inhibitor	3 (7.7)	4 (7.8)	1.000
Biologic agents	1 (2.6)	4 (7.8)	0.384

Patients were categorized into low-LDG (n = 39) and high-LDG (n = 51) groups using a cut-off of 2.21% (90th percentile of LDG levels in the HC group). Demographic, clinical, and laboratory parameters and treatments were compared between the two groups. Continuous variables are presented as median (interquartile range) and were compared using the Mann–Whitney U test; categorical variables are presented as n (%) and were compared using the chi-square (χ^2^) test. Fisher’s exact test was used when expected cell counts were <5. * *p* < 0.05, ** *p* < 0.01. Abbreviations: SjD, Sjögren’s disease; LDG, low-density granulocyte; HC, healthy control; ESSDAI, EULAR Sjögren’s Syndrome Disease Activity Index; ESR, erythrocyte sedimentation rate; JAK, Janus kinase.

## Data Availability

The data underlying this article will be shared on reasonable request to the corresponding author.

## Supplementary Materials

The following supporting information can be downloaded at: https://www.mdpi.com/article/10.3390/ijms27156722/s1.

## Abbreviations

The following abbreviations are used in this manuscript:

SjD	Sjögren’s disease
LDG(s)	Low-density granulocyte(s)
NDN(s)	Normal-density neutrophil(s)
HC(s)	Healthy control(s)
PBMC(s)	Peripheral blood mononuclear cell(s)
ESSDAI	EULAR Sjögren’s Syndrome Disease Activity Index
IL-6	Interleukin-6
TNF-α	Tumor necrosis factor-alpha
Th17	T helper 17
ESR	Erythrocyte sedimentation rate
MFI	Mean fluorescence intensity
ILD	Interstitial lung disease
KL-6	Krebs von den Lungen-6
SD	Standard deviation
IQR	Interquartile range
HCQ	Hydroxychloroquine
MMF	Mycophenolate mofetil
JAK	Janus kinase
CsA	Cyclosporin A
SLE	Systemic lupus erythematosus
LN	Lupus nephritis
DM	Dermatomyositis
NETosis	Neutrophil extracellular trap formation
MDSC(s)	Myeloid-derived suppressor cell(s)
PMN-MDSC(s)	Polymorphonuclear myeloid-derived suppressor cell(s)
NLR	Neutrophil-to-lymphocyte ratio
NPR	Neutrophil-to-platelet ratio
CTD	Connective tissue disease
GITRL	Glucocorticoid-induced tumor necrosis factor receptor-related ligand
AhR	Aryl hydrocarbon receptor
ESS	Experimental Sjögren’s syndrome
AECG	American–European Consensus Group
DLCO	Diffusing capacity for carbon monoxide
FVC	Forced vital capacity
LPS	Lipopolysaccharide
IFN(s)	Interferon(s)
