# Supplementary material for: Low-Density Granulocytes Link to Disease Activity, Organ Involvement, and Cytokine Production in Sjögren’s Disease
Source: Int J Mol Sci. 2026 Jul 28;27(15):6722. doi: 10.3390/ijms27156722 (PMC13466453; doi:10.3390/ijms27156722)
Supplement: Supplementary file 1 [file ijms-27-06722-s001.zip › ijms-4421995-supplementary.pdf]

SUPPLEMENTARY MATERIAL

Figure S1. Flow cytometry gating strategy for the identification of low-density granulocytes (LDGs).

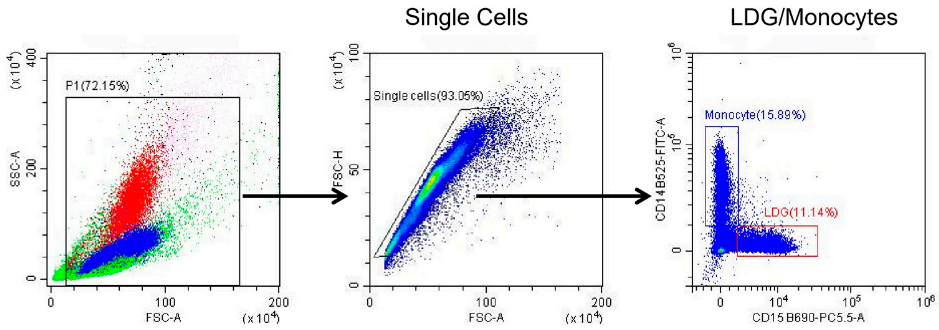

Figure S1. Representative flow cytometry gating strategy used for the identification of low-density granulocytes (LDGs) within the PBMC fraction. Cells were first gated according to forward scatter (FSC) and side scatter (SSC) characteristics to exclude debris, followed by singlet discrimination (FSC-A vs. FSC-H) to remove cell aggregates. LDGs were subsequently identified as $CD14^{-/low}CD15^{+}$ cells, while $CD14^{+}CD15^{-}$ cells were defined as monocytes. This gating strategy was applied consistently to all samples throughout the study.

Table S1. Clinical characteristics and treatment regimens of the longitudinal follow-up cohort

Patient	Medications during follow-up	ESSDAI Baseline	ESSDAI Follow-up	ΔESSDAI	LDG% Baseline	LDG% Follow-up	ΔLDG%
P1	GC+ RTX	7	5	-2.00	4.29	1.71	-2.58
P2	GC+ HCQ	7	7	0.00	6.16	4.83	-1.33
P3	MMF	10	10	0.00	7.68	1.34	-6.34
P4	HCQ+ MMF	6	4	-2.00	2.77	0.95	-1.82
P5	HCQ+ MMF	6	6	0.00	10.25	4.41	-5.84
P6	HCQ+ MMF	7	5	-2.00	3.5	0.43	-3.07
P7	MMF+ BEL	7	2	-5.00	3.89	2.91	-0.98
P8	GC+ HCQ	4	2	-2.00	1.9	1.47	-0.43
P9	GC+ HCQ+ CsA	2	2	0.00	1.98	0.76	-1.22
P10	HCQ+ CsA	0	0	0.00	3.31	0.35	-2.96
P11	HCQ	2	0	-2.00	3.42	2.13	-1.29

P12	HCQ+ GC+ MMF	7	5	-2.00	3.44	5.86	2.42
P13	HCQ+GC+RTX	14	10	-4.00	11.14	3.05	-8.09

Abbreviations: ESSDAI, EULAR Sjögren's Syndrome Disease Activity Index; LDG, low-density granulocyte; GC, glucocorticoids; HCQ, hydroxychloroquine; MMF, mycophenolate mofetil; RTX, rituximab; BEL, belimumab; CsA, cyclosporine A.
